# Long-Term Psychological Consequences of World War II Trauma Among Polish Survivors: A Mixed-Methods Study on the Role of Social Acknowledgment

**DOI:** 10.3389/fpsyg.2020.00210

**Published:** 2020-02-26

**Authors:** Marcin Rzeszutek, Maja Lis-Turlejska, Aleksandra Krajewska, Amelia Zawadzka, Michał Lewandowski, Szymon Szumiał

**Affiliations:** ^1^Faculty of Psychology, University of Warsaw, Warsaw, Poland; ^2^Faculty of Psychology, SWPS University of Social Sciences and Humanities, Warsaw, Poland

**Keywords:** World War II trauma, PTSD, depression, social acknowledgment, mixed-methods design

## Abstract

**Background:**

The research on the psychological consequences of World War II (WWII) trauma has predominantly focused on concentration camp and Holocaust survivors. Only a few studies have been undertaken among civilian survivors of WWII.

**Objectives:**

The purpose of this study was to examine the association between perceived social acknowledgment of WWII trauma and the level of post-traumatic stress disorder (PTSD) and depressive symptoms among Polish survivors of WWII by employing a mixed-methods design (i.e., a quantitative analysis supported by qualitative interviews).

**Method:**

In the quantitative part, 123 participants filled out: the list of WWII-related traumatic events, the PTSD Checklist for the Diagnostic and Statistical Manual of Mental Disorders, Fifth Edition (PCL-5), the shortened version of the Geriatric Depression Scale (GDS), and the Social Acknowledgment Questionnaire (SAQ). In the qualitative part, an interpretative phenomenological analysis (IPA) of participants’ reminiscences of WWII was examined.

**Results:**

Although we observed a direct positive association between the number of WWII-related traumatic events and the intensity of PTSD and depressive symptoms, these relationships changed when we entered the social acknowledgment construct into the model. Specifically, we found that perceived social acknowledgment (general disapproval) was a mediator of the relationship between the number of WWII traumatic events and the intensity of PTSD symptoms only, and not of depressive symptoms. In the qualitative part, three themes relating to traumatic reminiscences emerged among the participants: *parental efficacy, parental betrayal*, and *support from the invader*.

**Conclusion:**

Our study showed the significance of the general social acknowledgment in the long-term mental consequences of the WWII trauma in Poland. In addition, the results of our study may be an adjunct to the discussion on the long-term impact of WWII trauma in Poland and the factors that hindered its social recognition.

## Introduction

The research on the psychological consequences of World War II (WWII) began in Europe in the middle of the 20th century and initially focused predominantly on concentration camp and Holocaust survivors (e.g., Bastiaans, 1957; Krystal, 1968). In the late 1990s, an increasing number of authors also started to examine the psychological impact of WWII trauma among civilian populations, mainly investigating the prevalence of post-traumatic stress disorder (PTSD; e.g., Bramsen and van der Ploeg, 1999; Kuwert et al., 2007; Glaesmer et al., 2010; Glück et al., 2012). The above-mentioned authors admitted that the long-lasting influence of WWII trauma exposure may persist for decades, which was observed not only in studies conducted on convenience samples of WW-II survivors (e.g., 10.9% PTSD rate; Kuwert et al., 2007), but also among general population samples (e.g., 4.0% PTSD rate; Glaesmer et al., 2010).

Conversely, similar research programs carried out in Poland indicate a significantly higher PTSD levels among Polish survivors of WWII. For example, Lis-Turlejska et al. (2012, 2018) revealed that the PTSD rate in this population varied from 29.4 to 38.3%. Several historical factors may be responsible for such a high PTSD level in Poland, including, first and foremost, the tremendous human losses during WWII, which were the highest compared to all countries involved in the war (Davies, 2005). On the other hand, Polish people faced significant obstacles in coping with this kind of trauma after the end of WWII due to socio-political reasons. Namely, during the communist regime in Poland after the end of WWII, many Poles experienced repression and insecurity, which not only precluded revealing WWII traumatic experiences to other people, but even recognizing themselves as war victims (Davies, 2005). In other words, Polish survivors of WWII were not able to share their war-related trauma experiences in a stable, safe, and supportive community, which is attributed as a key factor in both PTSD treatment and prevention (Foa et al., 2005).

The afore-mentioned problem is now being dealt with in the most recent PTSD models, which have shifted away from the traditionally studied, individual risk factors for this disorder (e.g., Brewin et al., 2000) toward social and interpersonal variables (e.g., Maercker and Müller, 2004; Maercker and Horn, 2013; Maercker et al., 2016). In this regard, Maercker and Horn (2013) created the socio-interpersonal model of PTSD, where the central term is the *social acknowledgment of a trauma victim or survivor*, relating to the extent to which a trauma survivor perceives and feels social empathy and understanding of his/her traumatic experiences from an intimate partner, community or even from the whole of society. Alternatively speaking, trauma victims may either experience support from their close surroundings or negative reactions toward their experiences such as being ignored or even blamed for the trauma. Several studies have shown that social acknowledgment is negatively associated with PTSD symptoms among various populations post-trauma (e.g., Maercker and Müller, 2004; Mueller et al., 2009), a trend that is most apparent among war survivors (Schumm et al., 2014).

It is well established that PTSD is associated with many psychiatric comorbidities, of which depression is the most prevalent (e.g., Kessler et al., 1995; Flory and Yehuda, 2015). PTSD–depression comorbidity is especially visible in combat-related PTSD (Stander et al., 2014; Jasbi et al., 2018). Several explanations for this comorbidity are highlighted in the literature, including the fact that PTSD may be a causal risk factor for subsequent depressive disorders (e.g., Wright et al., 2011), the notion that PTSD and depression share similar risk vulnerabilities (e.g., O’Toole et al., 2009), or the hypothesis that the afore-mentioned comorbidity is a type of artifact due to symptoms overlapping in the diagnostic process (Biehn et al., 2013). However, it is not entirely known whether war-related trauma acts as a mutual risk factor for both PTSD and depression, as some authors have observed that this kind of trauma predicted both PTSD and depression (e.g., Hoge et al., 2006), while other researchers only found a link to PTSD (e.g., Larson et al., 2008). More recent studies have underlined the need to incorporate positive and negative social reactions toward this type of trauma in order to resolve this controversy (Schumm et al., 2014).

To make matters more complicated, one should also bear in mind that for WWII survivors, aging may modify this potential comorbidity (Kang et al., 2018). Specifically, age−related factors such as a decline in cognitive functioning, other physical comorbidities, and retirement, sometimes associated with poor social support, may all exacerbate the PTSD–depression association among older adults (Osei-Boamah et al., 2013). However, the research on the link between PTSD and depression for WWII-related trauma is still relatively scarce and relies predominantly on a quantitative methodological design (e.g., Trappler et al., 2007; Glaesmer et al., 2010). According to an increasing number of authors, relying solely on a quantitative analysis without qualitative interviews may limit obtaining a thorough picture of the level of coping linked to war-related trauma, especially when considering the long-lasting impact of traumatic memories (Creswell and Zhang, 2009).

## Current Study

**FIGURE 1:**
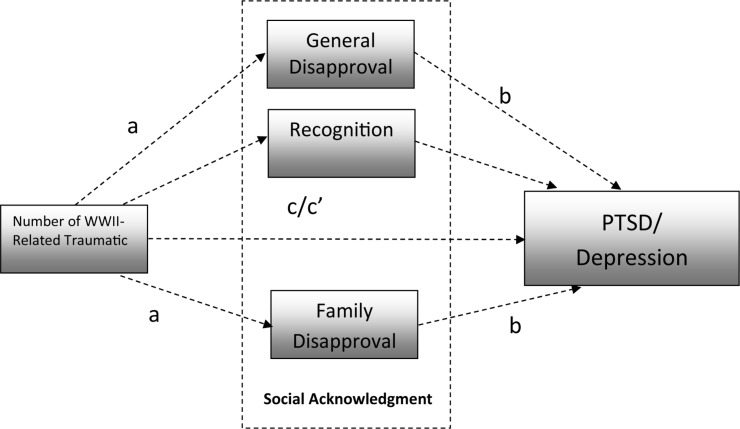
Hypothesized relationships between analyzed variables.

Taking the afore-mentioned research gaps into consideration, the purpose of this study was to examine the association between the perceived social acknowledgment of WWII trauma and the level of PTSD and depressive symptoms among Polish survivors of WWII using a mixed-methods design (i.e., using a quantitative analysis supported by qualitative interviews conducted among participants). More specifically, we wanted to investigate whether the number of traumatic events was related to the level of PTSD and depressive symptoms and whether this association was mediated by the social acknowledgment of trauma for WWII survivors (see Figure 1). In addition, we aimed to explore whether the above-mentioned relationship on the quantitative level could be strengthened by the qualitative analysis [i.e., an interpretative phenomenological analysis (IPA)]. We formulated six hypotheses:

**Hypothesis 1**. A positive relationship exists between the number of WWII-related traumatic events and the intensity of PTSD symptoms (from all clusters, B, C, D, E, as well as the total intensity of PTSD symptoms) among participants.
**Hypothesis 2**. A positive relationship exists between the number of WWII-related traumatic events and the intensity of depressive symptoms among participants.
**Hypothesis 3**. Perceived social acknowledgment (i.e., family disapproval, general disapproval, and recognition as a victim) of WWII trauma is negatively related to the level of PTSD symptoms among participants.
**Hypothesis 4**. Perceived social acknowledgment of WWII trauma is negatively related to the level of depressive symptoms among participants.
**Hypothesis 5**. The relationship between the number of WWII-related traumatic events and the intensity of PTSD symptoms is at least partially mediated by the perceived social acknowledgment of WWII trauma.
**Hypothesis 6**. The relationship between the number of WWII-related traumatic events and the intensity of depressive symptoms is also at least partially mediated by perceived social acknowledgment.

## Materials and Methods

### Participants and Procedure

The study was conducted on the convenience sample of the Polish WWII survivors and the participants were residents in various social welfare homes in Poland. The study was carried out by one clinical psychologist and psychotherapist, with the aid of two research assistants. After informed consent was obtained, the participants filled out a paper-and-pencil version of the questionnaires and took part in the study voluntarily, as there was no remuneration for participation. The inclusion criteria were a willingness to talk about their memories and experiences during WWII. The exclusion criteria encompassed signs of dementia, which were screened by the clinical psychologists employed in the welfare homes, where the research was carried out.

One hundred and seventy-five participants participated in the study: 114 females (65.1%) and 61 males (34.9%) aged 74–103 years old (*M* = 86.73; *SD* = 5.71). However, 52 participants were excluded from the final simple due to missing data in their answers to the administered questionnaires, which precluded the statistical analysis. Therefore, the final study sample consisted of 123 WWII survivors aged 74–103 years old (*M* = 82.53; *SD* = 5.74). Table 1 presents the study sample’s socio-medical characteristics for the final sample and for the participants with missing data, along with the values for the Pearson’s chi-squared test for independence and the Students’ *t*-test for independent samples, both of which were used to check if the final sample differed from the group of participants with missing data.

**TABLE 1:** Socio-medical variables in the studied final sample (*N* = 123) and in the missing data group (*N* = 52).

Variable	Final	Missing data	
	sample	group	
		
	*N* (%)	*N* (%)	Statistical test
**Gender**			
Male	41(33.3%)	32(61.5%)	χ^2^(1) = 0.42, *p* > 0.05
Female	82(66.7%)	20(38.5%)	
Age in years (*M* ± *SD*)	86.53 ± 5.74	87.19 ± 5.67	*t*(173) = -0.70, *p* > 0.05
**Marital status**			
Married	10(8.1%)	4(7.7%)	χ^2^(5) = 2.94, *p* > 0.05
Single	12(9.8%)	6(11.5%)	
Informal relationship	3(2.4%)	0(0%)	
Separated	1(0.8%)	0(0%)	
Divorced	10(8.1%)	7(13.5%)	
Widowed	87(70.7%)	35(67.3%)	
**Education**			
Elementary	24(19.5%)	7(13.5%)	χ^2^(4) = 2.80, *p* > 0.05
Vocational	13(10.6%)	6(11.5%)	
Secondary	52(42.3%)	22(42.3%)	
Not completed higher	3(2.4%)	0(0%)	
Higher education	31(25.2%)	17(32.7%)	

**M*, mean; *SD*, standard deviation; χ^2^, chi-squared test of independence; *t*, students’ test for independent samples; *p*, statistical significance.*

In the qualitative part of the study, of the final study sample of 123 individuals, 16 WWII survivors (three male and 13 female) agreed to take part in the interviews. The inclusion criteria were analogous to those used for the quantitative part of the study. The average age of these survivors was 85.2 (*SD* = 6.8), which means that they were on average 6–11 years old during WWII. Some of the participants (six) witnessed the Warsaw Uprising. None of them took an active part in the fighting nor were any of them prisoners in a concentration camp. Last but not least, the research design was approved by the local ethics committee.

The participants who were excluded from the analysis did not differ in terms of sex, age, education, or marital status. The size of the sample allowed for the detection of effects with a Cohen’s f2 value of at least 0.10 when a statistical power of 0.80 and a significance level of 0.05 were assumed. A Cohen’s f2 value of at least 0.10 indicates that the analysis was sensitive to small to medium effect sizes because an f2 of 0.02 is considered a small effect, and an f2 of 0.15 is considered a medium effect according to Cohen’s guidelines (Cohen, 1988).

### Measures

#### Quantitative Level

##### WWII-related traumatic events

The first part of the survey included a list of WWII-related traumatic events (Lis-Turlejska et al., 2016), where the participants had to answer whether they had directly experienced any of the 18 WWII traumatic events (e.g., torture, imprisonment in a Nazi concentration camp, rape, loss of one’s mother, bombing, extreme hunger) and whether they had witnessed any of the following six traumatic events (shooting of a person, execution, rape or any other sexual violence, heavy beating of a person, assault, persecution of Jews). The participants answered each item in a yes/no format. The sum of the “yes” answers was evaluated as a result (i.e., the number of WWII-related traumatic events).

##### Post-traumatic stress disorder (PTSD) symptoms

To assess the intensity of PTSD symptoms, the Polish adaptation of the PTSD Checklist for the Diagnostic and Statistical Manual of Mental Disorders, Fifth Edition (DSM-5, PCL-5; Weathers et al., 2013) was used in the Polish adaptation. The PCL-5 consists of 20 items created on the basis of a PTSD diagnosis in the DSM-5, where each scale represents some DSM-5 criteria (B-intrusions; C-avoidance; D-negative cognitions and mood alterations; E-hyperarousal and heightened reactivity; Weathers et al., 2013). Subjects indicate how much a particular symptom has affected them in the previous month on a five-point scale. Possible answers are: *not at all, slightly, moderately, much*, and *very strongly*. Cronbach’s α reliability coefficients for all scales in this study were satisfactory (see Table 2).

**TABLE 2:** Descriptive statistics and pearson correlation coefficients between analyzed variables.

Variables	α	*M*	*SD*	*S*	*K*	1	2	3	4	5	6	7	8	9
1. Number of traumatic events	–	6.71	4.13	0.30	–0.55	–								
2. General disapproval	0.77	6.73	4.40	0.04	–0.83	0.464**	–							
3. Recognition	0.70	3.06	3.16	0.90	0.16	0.190	0.057	–						
4. Family disapproval	0.71	4.63	3.31	0.75	0.72	–0.046	0.136	–0.157	–					
5. PTSD symptoms criterion B	0.82	5.21	5.59	0.83	–0.09	0.361**	0.498**	0.151	0.166	–				
6. PTSD symptoms criterion C	0.84	1.92	2.63	0.82	–0.08	0.248**	0.389**	0.010	0.292**	0.444**	–			
7. PTSD symptoms criterion D	0.73	7.12	5.42	0.48	–0.58	0.227*	0.562**	0.056	0.175	0.488**	0.301**	–		
8. PTSD symptoms criterion E	0.72	5.56	4.93	0.88	0.43	0.116	0.391**	0.186	0.115	0.476**	0.137	0.600**	–	
9. PTSD symptoms total	0.86	19.80	14.31	0.58	–0.45	0.313**	0.614**	0.142	0.226*	0.821**	0.519**	0.831**	0.783**	–
10. Depression	0.77	6.01	3.53	0.28	–0.68	0.227*	0.538**	–0.309	0.266**	0.381**	0.308**	0.477**	0.406**	0.525**

*M*, mean value; *SD*, standard deviation; *S*, skewness; *K*, kurtosis; *p < 0.05; **p < 0.01.

##### Depressive symptoms

To measure the severity of depressive symptoms among participants, a shortened version of the Polish adaptation of the Geriatric Depression Scale (GDS; Sheikh and Yesavage, 1986) was used in the Polish adaptation. The GDS serves as a screening tool for assessing the severity of depressive symptoms among old adults. The short GDS version consists of 15 questions with a yes/no answer option. Cronbach’s α reliability coefficients for all scales in this study were also satisfactory (see Table 2).

##### Perceived social acknowledgment

To examine the level of perceived social acknowledgment of WWII trauma, the Polish adaptation of the Social Acknowledgment Questionnaire (SAQ; Maercker and Müller, 2004) was used. The SAQ is a self-report scale with 16 items that consists of three subscales: family disapproval, general disapproval, and recognition as a victim. These scales refer to positive (e.g., recognition), as well as negative (e.g., rejection, disapproval) aspects of perceived social reactions in the social community following an experienced traumatic event. The respondents have to answer each statement using a four-point Likert scale: *I completely disagree, I agree a bit, agree to a large extent*, and *completely agree*. Cronbach’s α reliability coefficients for all scales in this study were satisfactory as well (see Table 2).

#### Qualitative Level

##### WWII-trauma related memories

In the qualitative part of the study, an IPA (Smith and Osborn, 2003) was used to explore individuals’ memories of childhood experiences from the WWII period. The research focused on the content and main themes of early-childhood memories with the aim of examining how those memories were related to elements of the social recognition of their trauma. The data were collected in the form of an interview that was undertaken simultaneously with the questionnaire from the quantitative part of the study. The interviews were carried out by a clinical psychologist, who was also a psychotherapist in order to take emotional care of the participants and to intervene if necessary (there was no need for any interventions). The interviews took place first before the questionnaire was filled in. This was done so as to avoid the influence of the questionnaire items’ content on the shape of their memories. These direct interviews lasted from 30–50 min. The interviewer minimized his or her influence on the content of the story. Therefore, apart from the preliminary question, *Please tell me about your experiences from* WWII, further interventions were avoided, and the interviews were limited to active listening and shifting the participants’ focus back to wartime if they started discussing later periods of their life.

### Data Analysis

In this study, a *mixed-methods design* was employed to examine our research hypotheses, a design that has been postulated as a way of enriching trauma research in general, traditionally dominated by quantitative data collection only (Creswell and Zhang, 2009). More specifically, in our study, a *concurrent design* was used, as we collected both quantitative and qualitative data parallel (Creswell, 2014). Both sources of data were then analyzed separately, and then mixed in accordance with side-by-side comparison, so the distinctiveness of each databases dissolved in the results and discussion section (Creswell and Zhang, 2009). The main idea of this design is to understand a given research topic from the perspective of two different types of evidence, quantitative and qualitative (*ibidem*).

The quantitative statistical analysis comprised firstly of descriptive statistics and Pearson’s correlation coefficients between the analyzed variables. The principal part of the analysis was performed with PROCESS macro software based on Model No. 4, which allows for multiple mediators to be tested: one explanatory variable and one explained variable (Hayes, 2018). The investigated model comprised three parallel mediators: general disapproval, recognition, and family disapproval, with five explained variables—the PTSD criteria from B to E and the level of depression—which were tested in five different mediation models. The explanatory variable in each model was the number of WWII-related traumatic events. All regression coefficients were acquired with the use of the mediation analysis. Hypothesis H1 was verified with the use of regression coefficients testing the relationship between the number of traumatic events and the intensity of PTSD symptoms (path c). Hypothesis H2 was verified with the use of regression coefficients testing the relationship between the number of traumatic events and the intensity of PTSD symptoms (path c). Hypotheses H3 and H4 were verified with the use of regression coefficients testing the relationship between social acknowledgment and the intensity of PTSD symptoms and depressive symptoms, respectively. The mediation effects were tested through a test based on bootstrap sampling, with the number of bootstrap samples equal to 5000. In mediation analysis, bootstrapping is used to generate the sampling distribution of the indirect effect and provides inferences that are more accurate than when the normal theory approach is used. A statistically significant total effect of the relationship between the number of traumatic events and PTSD and depression was not considered an indispensable condition for the detection of mediation, because opposing mediational processes can conceal the relationship between explanatory and explained variables and a test of the mediated effect has more statistical power than the test of the overall relation between the two (MacKinnon, 2008a, b). Full mediation was detected, when the direct relationship between the number of WWII-related traumatic events and PTSD symptoms was statistically insignificant after a mediator was included in the model. Partial mediation was to be detected, when after the inclusion of a mediator the direct relationship between the number of WWII-related traumatic events and PTSD symptoms was still statistically insignificant. In this way, the hypotheses H5 and H6 were verified.

The qualitative interview transcripts were analyzed using the principles of IPA (Smith and Osborn, 2003). To begin the process, the first transcript was read with initial insights being annotated. At the next stage of the analysis, a translation of these initial notes into emergent themes at a higher level of abstraction was undertaken. Then, the themes were examined to make conceptual links among them and related themes were clustered together. This process was repeated for each transcript using the superordinate themes from the first participant and identifying additional ones. After the analysis had been conducted on each transcript, patterns were established across participants and documented into a list of themes.

## Results

Table 2 presents the descriptive statistics for all analyzed interval variables.

The skewness and kurtosis values fell between -1 and 1, so the use of parametric statistical methods was appropriate. The Pearson’s correlation coefficients revealed positive relationships between the number of WWII-related traumatic events and the intensity of PTSD symptoms (for all diagnostic criteria with the exception of criterion E) and depressive symptoms. The number of WWII-related traumatic events also positively correlated with general disapproval. General disapproval also positively correlated with the intensity of the PTSD (for all diagnostic criteria) and depressive symptoms. Recognition did not correlate with PTSD nor with depressive symptoms. Family disapproval positively correlated with PTSD symptoms from criterion C, the total intensity of the PTSD symptoms, and the level of depressive symptoms. The level of depressive symptoms positively correlated with the intensity of PTSD symptoms (for all criteria) and the intensity of PTSD symptoms from different criteria positively intercorrelated, with the exception of the intensity of PTSD symptoms from criteria C and E, which did not correlate with each other. Twenty-five participants (20.3%) fulfilled the DSM-5 diagnostic criteria for PTSD. Sixty-six participants (53.7%) had scores suggesting depression (GDS total score > 5).

Table 3 presents the types and number of WWII-related traumatic events that the participants were exposed to.

**TABLE 3:** Frequency distribution – types of WW-II-related traumatic events.

	*N*	%
Type of WW-II related traumatic event		
Loss of one’s mother	9	7.3
Loss of one’s father	22	17.9
Loss of one’s close relative	50	40.7
Being in combat	11	8.9
Being in resistance	10	8.1
Being wounded	16	13.0
Killed someone	6	4.9
Being tortured	9	7.3
Being imprisoned in a Nazi concentration camp	8	6.5
Being imprisoned in a Soviet camp	8	6.5
Being in a ghetto	3	2.4
Being in Warsaw during the Warsaw uprising	47	38.2
Participated in the Warsaw uprising	14	11.4
Experienced rape or other form of sexual abuse	2	1.6
Surviving bombing	95	77.2
Had to remain in hiding	67	54.5
Hiding Jews	19	15.4
Being forcedly relocated to Siberia	4	3.3
Being in forced labor	21	17.1
Health or life threatening cold	57	46.3
Life threatening hunger	71	57.7
Witnessed combat	58	47.2
Witnessed somebody being shot	61	49.6
Witnessed execution or murder	56	45.5
Witnessed rape or other form of sexual abuse	9	7.3
Witnessed somebody being heavily beaten	50	40.7
Witnessed assault or persecution of Jews	42	34.1

The majority of participants were exposed to bombing. Experiencing rape or other forms of sexual abuse was the least frequent event. To test the formulated hypotheses, regression coefficients, computed with macro Process for the mediation models, were used (Hayes, 2018). The results are presented in Table 4.

**TABLE 4:** The results of analysis of relationships between the number of WWII-related traumatic events, PTSD, and depressive symptoms mediated by perceived social acknowledgment.

		Relationships						
		
Mediator	Explained variable	Path a	Path b	Path c	Path c’	Indirect effect	Df	*R*^2^
General disapproval	PTSD symptoms criterion B	0.43*	0.75*	0.52	−0.12	0.01÷0.91	4,118	0.30
	PTSD symptoms criterion C	0.43*	0.24	0.15	0.04	−0.02÷0.28	4,118	0.15
	PTSD symptoms criterion D	0.43*	1.12**	0.36	−0.12	0.02÷1.09	4,118	0.44
	PTSD symptoms criterion E	0.43*	0.45	0.40	0.13	−0.02÷0.71	4,118	0.18
	PTSD symptoms total	0.43*	2.75**	1.43	0.17	0.05÷2.77	4,118	0.18
	Depressive symptoms	0.43*	0.49**	0.31	0.14	−0.02÷0.46	4,118	0.42
Recognition	PTSD symptoms criterion B	0.20	0.24	0.52	−0.12	−0.10÷0.31	4,118	0.30
	PTSD symptoms criterion C	0.20	0.01	0.15	0.04	−0.07÷0.11	4,118	0.15
	PTSD symptoms criterion D	0.20	0.03	0.36	−0.12	−0.15÷0.20	4,118	0.44
	PTSD symptoms criterion E	0.20	0.27	0.40	0.13	−0.11÷0.31	4,118	0.18
	PTSD symptoms total	0.20	0.54	1.43	0.17	−0.24÷0.74	4,118	0.18
	Depressive symptoms	0.20	−0.32*	0.31	0.14	−0.18÷0.07	4,118	0.42
Family disapproval	PTSD symptoms criterion B	0.08	0.27	0.52	−0.12	−0.10÷0.36	4,118	0.30
	PTSD symptoms criterion C	0.08	0.14	0.15	0.04	−0.05÷0.11	4,118	0.15
	PTSD symptoms criterion D	0.08	−0.14	0.36	−0.12	−0.12÷0.29	4,118	0.44
	PTSD symptoms criterion E	0.08	0.20	0.40	0.13	−0.13÷0.34	4,118	0.18
	PTSD Symptoms Total	0.08	0.47	1.43	0.17	−0.24÷0.97	4,118	0.18
	Depressive symptoms	0.08	0.39*	0.31	0.14	−0.10÷0.20	4,118	0.42

df, degrees of freedom; *R*^2^, variance explained. Number of WW-related traumatic events was the explained variable in each model. *p < 0.05; **p < 0.01.

There were statistically significant positive correlations between the number of WWII-related traumatic events and the intensity of PTSD and depression symptoms (see Table 2), which is consistent with H1 and H2. However, there were no statistically significant relationships between the number of WWII-related traumatic events and the intensity of the symptoms of depression in the statistical models that contained both the number of WWII-related traumatic events and perceived social acknowledgment (paths c and c’). The statistically significant relationships between general disapproval and the intensity of the PTSD symptoms from the B and D criteria and the total intensity of the PTSD symptoms were consistent with H3. Nevertheless, the other dimensions of perceived social acknowledgment (i.e., recognition and family disapproval) were not significantly related to PTSD symptoms.

Relationships between the level of depressive symptoms and general disapproval and between the level of depressive symptoms and family disapproval were both statistically significant and positive, while the relationship between depressive symptoms and recognition was negative. These results are in line with H4.

General disapproval mediated the relationship between the number of traumatic events and the total intensity of the PTSD symptoms. To investigate the matter further, specifically to see if the mediation effect concerns all of the PTSD symptoms clusters or only some of the analysis was also performed in the models containing specific symptoms clusters. General disapproval mediated the relationship between the number of traumatic events and the total intensity of the PTSD symptoms from criteria B and D. These results are consistent with H5. However, it was a full mediator, because the direct relationship between the number of WWII-related traumatic events and PTSD symptoms was statistically insignificant (path c’).

There were no statistically significant mediation effects regarding depressive symptoms. Social acknowledgment was not a mediator of the relationship between the number of WWII traumatic events and depressive symptoms, so there were no results supporting H6; however, as mentioned above, all three dimensions of social acknowledgment were statistically related to depressive symptoms.

For the further elaboration of the acquired results, qualitative methods were applied. We examined meanings of autobiographical stories told by the participants during direct interviews. The thematic analysis of this data suggests three overarching themes of reminiscence: *parental efficacy, parental betrayal*, and *support from the invader*. *Parental efficacy* theme contains stories related to parents’ behavior in threatening situations, such as avoiding danger as a result of the parents’ actions, support from the parents toward the child in a threatening situation, and support from the parents toward other people in a threatening situation. Parents here are seen as coping, competent, effective, and giving support. “Fortunately, my father knew German and began to explain to the Gestapo that I was only a child, I am not dangerous and I did not mean anything bad. The Gestapo officer dropped his rifle down.”—describes one of the participants. *Parental betrayal* was related to difficult emotions relayed by the participants while telling stories about conscious exposure to danger, lack of concern in the face of danger, and abandonment in threatening situations. That *betrayal* could be deliberate (“My father gave me a bubble of milk and told me to go. I didn’t know what is going on. Later I realized that I was supposed to be bait for a German patrol.”) or random (“My parents sent me to my uncle for a few days. There, I heard that there is a war and I saw planes flying over the buildings… I got a panic attack and they could not calm me down. I was calling my mom and dad, and there was no contact with them for many days). The common and important factor was the high level of difficult emotions when telling these stories (“[crying] I felt so lonely. I have the impression that afterward I felt like this throughout my whole life.”). *Support from the invader* contained reminiscences about experiencing the “human” face of Nazis (e.g., offering material support, emotional support, or rescue in the face of danger). “He (German soldier) saw us walking along with others. He came to our parents. He told us to spread and walk separately, to share their children and say they are the only caregivers. He said he also has children at that age. Thanks to him we did not get to the camp.”

## Discussion

The results of our study were in line with the first two hypotheses, as we observed a direct positive relationship between the number of WWII-related traumatic events and the intensity of the PTSD and depressive symptoms among participants. On the one hand, when solely analyzing this finding and, for the moment, leaving aside the construct of social acknowledgment (see below), it is in line with the traditional *dose–response* models for both PTSD (Johnson and Thompson, 2008) and depression (Stander et al., 2014). On the other hand, it is worth underlining that a very high percentage of participants fulfilled the diagnostic criteria for PTSD (20.3%) as well as for clinical depression (53.7%), which is again much higher compared to other European countries with respect to the PTSD rate (e.g., Glaesmer et al., 2010) as well as the depression level (e.g., Trappler et al., 2007) in this type of population. In discussing this extraordinarily high PTSD and depression rate among Polish WWII survivors, which has also been noted in other studies (Lis-Turlejska et al., 2012), one cannot escape from the huge scale of human loss and terror (Snyder, 2010), nor from the socio-political conditions of life immediately after the war (1945–1947 years), and in Poland there were further battles to come. In addition, during the time of the Soviet regime, many Polish people, especially in the years 1946–1956, were treated as political enemies and experienced a high level of persecution, which can be viewed as continued traumatization after the initial WWII trauma (Snyder, 2010). In other words, great human losses during WWII, the constant atmosphere of threats, and repression during the Soviet regime after WWII, along with conditions that made it difficult for them to share their war-related trauma in their community explain the high level of psychopathology among Polish survivors of WWII (Lis-Turlejska et al., 2018).

However, the relationship between WWII trauma, PTSD, and depressive symptoms appeared to be much more complex when we entered the construct of social acknowledgment into the model. Namely, although we generally found evidence supporting H3 and H4 (see Table 3), we observed that perceived social acknowledgment (general disapproval) was only a mediator of the relationship between the number of WWII traumatic events and the intensity of PTSD symptoms (H5), and not of depressive symptoms (H6). In other words, it seems that out of two possible reactions to traumatic events (i.e., PTSD or depression), PTSD may be associated with lack of social recognition (see general disapproval), which in turn is related to the number of traumatic events. This finding corresponds to other studies conducted employing the socio-interpersonal model of PTSD (e.g., Mueller et al., 2009; Schumm et al., 2014). Conversely, although we observed a correlation between the number of WWII traumatic events and depressive symptoms, this link was not mediated by perceived social acknowledgment, which was opposed to H6. Alternatively speaking, the link between the number of WWII traumatic events and depressive symptoms disappeared after controlling for the dimensions of social acknowledgment, thus participants who experienced social acknowledgment were not found to be depressed, regardless of the degree of WWII traumatization. The aforementioned finding may enforce Maercker and Horn’s (2013) socio-interpersonal PTSD model, which clearly highlights the role of social responses in coping with traumatic experiences. In addition, our results may add somewhat to the discussion on PTSD–depression comorbidity (Stander et al., 2014). Specifically, it seems that other processes occur in the case of PTSD and yet others in the case of depression when we take into account the socio-interpersonal factors, a topic that certainly requires further investigation.

Finally, in this study, we conducted a qualitative follow-up to a quantitative analysis in order to use qualitative data to more deeply explain the results on the quantitative level (Creswell and Zhang, 2009). The interviews were focused on the content and main themes of early-childhood memories and were conducted with the assumption proposed by studies on autobiographical memory among Holocaust survivors that “Holocaust survivors remember what they remember because these memories have functional value” (Canham et al., 2017, p. 1159). A thematic analysis of these data showed three themes of reminiscence among participants: *parental efficacy, parental betrayal*, and *support from the invader*. It is well documented that autobiographical memory may foster identity and a sense of coherence, but at the same time, it may result in apathy, an absence of purpose and dwelling on past conflicts and losses (Webster and Haight, 2002; Cappeliez et al., 2005). In our study, two of the three main themes of reminiscence concern issues connected with parental attitudes and behaviors: in a positive sense, the efficacy of those caregivers, and in a negative sense, “betrayal” by those caregivers. On the one hand, situations involving parental agency may be an element of support and parental resilience, and are part of the war narrative of the entire family system. The events classified as *support from the invader* could have a similar potential to be a narrative of the family system (i.e., we can assume that they were an element that was mentioned and discussed by many members of the family system). On the other hand, a very vivid theme of *parental betrayal* supports the results of the quantitative part of the study in that family disapproval is a mediator of the WWII trauma–depression relationship. More specifically, these situations showed the fragility of the entire family system and we can assume that in such a context there is a greater likelihood of blaming the victim or of ostracism from the caregivers. In addition, these situations burden the caregivers, making it more difficult for our participants to acknowledge WWII trauma. We can also assume that suffering as a result of *parental betrayal*, as an individual trauma, could attract less social recognition than the suffering caused by the Nazi invaders, which is classified as a collective trauma (Canham et al., 2017). Obviously, our qualitative results need further examination but can shed some new light on the long-lasting psychological impact of WWII trauma.

### Strengths and Limitations

This study has several strengths, mostly including accessing a sample of WWII survivors and our use of a mixed-methods design, which has very rarely been used in trauma research. However, some limitations should also be emphasized. First, although our research was conducted in various regions of Poland, we used a convenience sample of WWII survivors collected from welfare homes, which could be associated with some sample bias. Specifically, this latter issue may be one of the possible explanations for the very high PTSD and depression rate observed in this study, but also found by the other authors, who examined this topic also on convenience samples only (e.g., Lis-Turlejska et al., 2012, 2018). Thus, research among general population samples of WWII survivors is needed to possibly replicate these findings. In addition, the sample size in the qualitative part was relatively small, so we should be cautious with the final conclusions obtained from this part of analysis. Second, although we excluded participants with dementia, we did not screen for individual differences with respect to the cognitive abilities of the participants, which could be responsible for the relatively high level of missing data among our participants, who were then not included in the main analysis. This particular aspect is especially crucial taking into an account the fact that not all PTSD symptoms occurred to be significantly related with WWII-relate traumatic events (e.g., lack of relationship between PTSD from E criterion representing hyperarousal and hyperactivity; Kang et al., 2018). Third, in our study, we concentrated on assessment of WWII traumatic events only, so we did not measure other traumatic events across the lifespan of participants. Future studies could control this latter aspect among the samples of WWII survivors. Finally, the retrospective assessment of WWII traumatic experiences, i.e., decades later the reproduction of trauma content, might have possible confounded the self-reported traumatic and depressive symptoms declared by our participants.

## Conclusion

Our study showed the significance of the general social acknowledgment in the long-term mental consequences of the WWII trauma in Poland. In addition, the results of our study may be an adjunct to the discussion on the long-term impact of WWII trauma in Poland and the factors that hindered its social recognition. Finally, it seems that providing some kind of psycho-education focused on the processing of WWII trauma survivors may be very important in Poland, especially in the light of positive experiences of other countries with this specific problem (e.g., Forstmeier et al., 2014; Knaevelsrud et al., 2014).

## Data Availability Statement

Data are available upon request to the corresponding author.

## Ethics Statement

The studies involving human participants were reviewed and approved by the Ethics Committee of the Faculty of Psychology, SWPS University of Social Science and Humanities in Warsaw. Written informed consent was obtained from the participant for the publication of any potentially identifiable images or data included in this article.

## Author Contributions

MR conceived and designed the study, supervised the data collection both in quantitative and qualitative part of the project, conducted the interpretation of the data, prepared drafted and revised version of the manuscript. ML-T participated in designing the study and the interpretation of the data, helped in preparing the drafted and revised version of the manuscript. AK and AZ collected the data in the quantitative part of the project and approved the submitted version of the manuscript. ML collected and interpreted the qualitative data and merged it with the quantitative data. SS has major contribution in statistical analysis in the quantitative part of the project.

## Conflict of Interest

The authors declare that the research was conducted in the absence of any commercial or financial relationships that could be construed as a potential conflict of interest.
